# Prospect of vasoactive intestinal peptide therapy for COPD/PAH and asthma: a review

**DOI:** 10.1186/1465-9921-12-45

**Published:** 2011-04-11

**Authors:** Dongmei Wu, Dongwon Lee, Yong Kiel Sung

**Affiliations:** 1 Department of Research, Mount Sinai Medical Center, Miami Beach, FL 33140, USA; 2 WCU program, Department of BIN Fusion Technology, Chonbuk National University, Korea; 3 ReSEAT Program, KISTI, 206-9 Cheongnyangni-dong, Dongdaemun-gu, Seoul 130-742, Korea; Department of Chemistry, Dongguk University, Phil-dong, Chung-gu, Seoul 100-715, Korea

## Abstract

There is mounting evidence that pulmonary arterial hypertension (PAH), asthma and chronic obstructive pulmonary disease (COPD) share important pathological features, including inflammation, smooth muscle contraction and remodeling. No existing drug provides the combined potential advantages of reducing vascular- and bronchial-constriction, and anti-inflammation. Vasoactive intestinal peptide (VIP) is widely expressed throughout the cardiopulmonary system and exerts a variety of biological actions, including potent vascular and airway dilatory actions, potent anti-inflammatory actions, improving blood circulation to the heart and lung, and modulation of airway secretions. VIP has emerged as a promising drug candidate for the treatment of cardiopulmonary disorders such as PAH, asthma, and COPD. Clinical application of VIP has been limited in the past for a number of reasons, including its short plasma half-life and difficulty in administration routes. The development of long-acting VIP analogues, in combination with appropriate drug delivery systems, may provide clinically useful agents for the treatment of PAH, asthma, and COPD. This article reviews the physiological significance of VIP in cardiopulmonary system and the therapeutic potential of VIP-based agents in the treatment of pulmonary diseases.

## 1. Introduction

Vasoactive intestinal peptide (VIP) is a 28-amino-acid peptide, which was first isolated from upper intestine, and has been characterized as a vasodilatory peptide [1]. VIP has a very widespread distribution in the central and peripheral nervous systems [2]. It is one of the most abundant neuropeptides found in the cardiovascular system and airways [2-5]. This neuropeptide exerts a wide range of biological actions, such as positive inotropic and chronotropic effects, pulmonary and coronary vasodilatation, bronchodilation, and anti-inflammatory effects, and thus it influences many aspects of cardiopulmonary function [6-8]. Studies using VIP deficient animals and using animal models of diseases have indicated that VIP has significant therapeutic potential in the treatment of cardiopulmonary diseases, including pulmonary arterial hypertension (PAH), chronic obstructive pulmonary disease (COPD) and asthma [9-11].

### Clinical manifestation of PAH

PAH is a disabling chronic disorder of the pulmonary vasculature, which is characterized by abnormal pulmonary vascular proliferation and remodeling, vasoconstriction, perivascular inflammation, and thrombosis, leading to elevated pulmonary arterial pressure, increases in peripheral vascular resistance, and it ultimately results in right heart failure and death [12,13]. The past two decades have seen significant advances with the development and clinical implementation of a number of medications for the treatment of PAH: prostanoids, endothelin-1 receptor antagonists, and phosphodiesterase type 5 inhibitors. However, the results remain unsatisfactory, with persistent high mortality, insufficient clinical improvement and no convincing report of any reversal of the disease process [12,13]. In addition, the current PAH therapy requires a cocktail of drugs to manage PAH symptoms and often leads to drug intolerance [14]. Therefore, it is necessary to develop additional novel therapeutic approaches that target the various components of this multifactorial disease. VIP provides the combined potential advantages of lowering pulmonary arterial pressure, improving blood circulation to the heart and lung, reducing inflammation of the heart and lung tissues, and is readily accepted by the body because it is natural to it [1-8]. Based on its multiple biological actions, the development of controlled release airway drug-delivery system with VIP has emerged as a novel therapeutic strategy for the treatment of PAH.

### Clinical manifestation of COPD and asthma

Chronic inflammatory airway diseases such as bronchial asthma or COPD are major contributors to the global burden of disease. COPD is characterized by a chronic, slowly progressive airway disorder resulting from a combination of pulmonary emphysema and irreversible reduction in the caliber of the small airways of the lung, resulting in airflow limitation [15]. Asthma is a complex, persistent, inflammatory disease characterized by airway hyperresponsiveness in association with airway inflammation. Although there are many allopathic treatments, including bronchodilators and corticosteroids, there is no single medication that is effective against both the inflammatory and bronchoconstrictive components of asthma [16]. VIP exerts functions not only as a vasodilator and bronchodilator but also as a potent immunomodulator [1,7,8], thus VIP has significant therapeutic potential in the treatment of pulmonary diseases, including: PAH, asthma and COPD. However, VIP-based drugs are not yet in clinical use, possibly because the poor metabolic stability and difficulty in administration routes. The development of long-acting VIP analogues, in combination with appropriate drug delivery systems, may provide clinically useful agents for the treatment of PAH/asthma/COPD. This article reviews the physiological significance of VIP in cardiopulmonary system and the therapeutic potential of VIP-based agents in the treatment of pulmonary diseases.

## 2. Expression and distribution of VIP in cardiovascular-pulmonary system

VIP is co-localized with acetylcholine in postganglionic parasympathetic neurons in the cardiovascular and respiratory systems [17]. In the mammalian heart, VIP was found in nerve fibers associated with atrial and ventricular myocardium, conduction system, and coronary vessels [18-21]. Immunofluorescent and radioimmunoassay studies have localized VIP to neuronal cell bodies of the intrinsic cardiac ganglia, axons and dendrites, and presynaptic nerve terminals from which VIP is released as a nonadrenergic-noncholinergic neurotransmitter [22]. In the peripheral nervous system, VIP is present in sympathetic ganglia, the vagus nerves, some motor nerves such as the sciatic nerve, autonomic nerves that supply exocrine glands, vascular and nonvascular smooth muscle, and ganglion-like clusters of neuronal cell bodies that provide 'intrinsic' organ innervation [18,23].

VIP is abundantly present in normal human lungs [1,2,24]. VIP-immunoreactivity (IR)-containing cells are present in the tracheobronchial smooth muscle layer and glands of airways, and within the walls of pulmonary and bronchial vessels [25,26]. VIP-IR nerve fibers are found as branching networks in the respiratory tract [4]. The frequency of these VIP-ergic fibers decreases as the airways become smaller, and only a few VIP-ergic fibers are present in bronchioles and alveolar space [26]. The pattern of VIP-ergic nerve fiber distribution largely follows that of cholinergic nerves, which is consistent with the colocalization of VIP with acetylcholine [27]. VIP is also co-localized with nitric oxide synthase (NOS) in human and guinea-pig airways [28-30]. In human airways, a co-localized immunoreactivity of VIP and NOS is found in airway intrinsic neuronal perikarya [28,30]. Furthermore, VIP has also been identified in some sensory nerves, including sub-epithelial airway nerves [27,31]; as well as in immune cells such as mast cells [32], eosinophils [33,34], and in different mononuclear cells and polymorphonuclear leukocytes [35]. A deficiency of VIP in the respiratory system is considered to be a pathogenetic factor in pulmonary disease [36,37].

## 3. VIP release and metabolism

Circulating VIP in men is found in low plasma levels. However, an increase in plasma concentration has been detected in conditions, such as gastrointestinal stimulation, during strenuous exercise, acute myocardial infarction and gastrointestinal tumors [38-41]. Circulating VIP is produced from VIP-containing nerve fibers. Many VIP-containing nerves have a perivascular distribution and it thus seems likely that VIP can exert important local effects without producing a detectable increase in systemic levels [42]. Myocardial blood vessels and also pulmonary blood vessels are innervated by VIP immunoreactive nerve fibers, which cause vascular smooth muscle dilation [18,23]. Endogenous VIP is released by high frequency nerve stimulation and also is released by neostigmine, as well as by serotonin, dopaminergic agonists such as bromocriptine and apomorphine, prostaglandins (PGE, PGD) and nerve growth factor [43,44].

Under physiological conditions, VIP is mainly cleaved by endopeptidase, whereas in states of airway inflammation, mast cell enzymes dominate the degradation of VIP [45-47]. VIP is readily degraded by enzymes, including neutral endopeptidase, mast cell-derived tryptase and chymase, thus preventing it from relaxing vascular or tracheal smooth muscle [45-47].

## 4. VIP receptors in cardiovascular-pulmonary system

The biological effects of VIP are mediated by two type II G-protein-coupled receptors: VPAC1 and VPAC2 [48]. Stimulation of VPAC receptors by VIP causes dose-dependent activation of adenylate cyclase, which increases cAMP concentrations, and activates cAMP- and cGMP-dependent protein kinases and leads to smooth muscle relaxation via decreasing intracellular calcium levels [49]. While VIP binds both VPAC1 and VPAC2 receptors with high affinity, VIP can also bind with low affinity to the pituitary adenylate cyclase activating peptide (PACAP) receptor. PACAP is another secretin family member peptide that exhibits extensive similarities to VIP and shares VIP receptors and functions [50].

High densities of VIP binding sites were found in the pulmonary vascular smooth muscle layer and in airway smooth muscle of large, but not smaller airways. VIP binding sites are also present in sub-mucosal glands, airway epithelium and in alveolar walls [24,51]. In the human upper respiratory tract, VIP receptors were found on submucosal glands, epithelial cells, and arterial but not sinusoidal vessels [5]. VIP receptors are also expressed in innate immune cell types, including human mast cells, neutrophils, and peripheral blood monocytes, and murine macrophages and dendritic cells [52-56]. VIP is thought to play a role in regulating immunity and inflammation. Studies using VPAC2 receptor knockout mice and transgenic mice overexpressing the VPAC2 receptor have revealed that the receptor regulates the balance between T-helper type 1 and 2 lymphocytes (Th1 and Th2 cells) by stimulating production of more Th2-type cytokines, which mediate hypersensitivity reactions (e.g. allergy) [57,58]. Thus, this receptor is believed to play an important functional role in the respiratory tract by regulation of immune effects of VIP in allergic diseases such as allergic bronchial asthma.

The wide spread presence of VIP receptors in a variety of tissues and organ systems has led to the potential limitation of its clinical application. Intravenous administration of VIP has been shown to ameliorate histamine-induced bronchoconstriction in asthmatic subjects; while it also caused cardiovascular side effects by decreasing systemic blood pressure, inducing tachycardia and cutaneous flushing [59]. Thus, the development of effective drug delivery systems with airway delivery capability for VIP-based respiratory therapy represents a possible therapeutic strategy.

## 5. Role of VIP in heart and blood vessels

VIP is a potent vasodilator in coronary and pulmonary blood vessels, as well as other systemic blood vessels. The presence of VIP nerve fibers and their receptors in the coronary and pulmonary arteries strongly suggests that this peptide is important in the regulation of cardiopulmonary blood flow. VIP induces endothelium-independent relaxation in most of the vascular beds, including cat cerebral artery, dog isolated carotid artery, pig coronary artery, and bovine pulmonary artery [3-6].

There is direct evidence that VIP acts on heart muscle in various experimental system. VIP exerts a primary positive inotropic effect on cardiac muscle. In dogs, VIP infusion increases cardiac contractility and improves ventricular-vascular coupling, thus VIP enhances delivery of mechanical energy from the LV to the circulatory bed [60]. In isolated atrial or ventricular muscle, VIP, increases developed isometric force and is greater than isoproterenol in enhancing ventricular muscle contractile force [61]. VIP also exerts a primary positive chronotropic effect in the heart. Injection of VIP directly into the dog sinoatrial artery increases heart rate by 37%, VIP also dose-dependently shortens the atrioventricular conduction time, decreases the atrial and ventricular refractory periods [61,62]. Endogenously released VIP increases atrial and ventricular contractility, and heart rate. Stimulation of the parasympathetic (vagal) nerves, during muscarinic and β-adrenergic receptor blockade in dogs, increases the atrial contractile force by 32%, increases heart rate by 37%, and also increases right ventricular contraction *and *relaxation by 28 and 33%, respectively [63,64]. In patients with acute myocardial infarction, the VIP concentration in the plasma may increase by 33-62% within 6 h of the onset of symptoms [41]. Upon acute coronary ischemia, VIP is released from neurons in the coronary vessels and myocardium, and may also be released from the splanchnic viscera, and can act as a vasodilator to reduce myocardial ischemia [18,65].

## 6. Biological actions of VIP in airway

VIP is a potent vasodilator of airway smooth muscle *in vitro *and *in vivo*. In isolated tracheal or bronchial segments, VIP attenuates the constrictor effect of histamine, prostaglandine F2α, endothelin, leukotriene D4, kallikrein and neurokinin A [66,67]. The bronchodilatory effect of VIP in human bronchi is almost 100 times more potent than adrenergic dilatation by isoproterenol, and VIP is the most potent endogenous bronchodilator described so far [68]. VIP is also involved in the regulation of airway mucus secretion. High density VIP-expressing nerve fibers and VPAC2 mRNA have been found in airway submucosal glands [25,69]. The role of VIP in airway mucus secretion has been controversial. VIP has been shown to have both stimulation and inhibition effects on airway secretion. In the human trachea, VIP inhibited methacholine-stimulated release of glycoproteins and lysozyme [70]. In the upper airways, VIP was shown to stimulate lactoferrin secretion from human nasal mucosal cells, but had little effects on mucous glycoprotein release [71]. VIP inhibits cholinergic secretion in ferret trachea, whereas it stimulates cholinergic secretion in the cat trachea [72,73]. Therefore, the importance of VIP in airway mucus secretion appears to differ from species to markers examined. Future studies using human tissue and cells need to be performed in order to further elucidate the role of VIP on mucus secretion that associated with hypersecretory diseases such as COPD or asthma.

## 7. VIP in inflammatory response

Progressive pulmonary inflammation is the hallmark of airway diseases, including asthma, COPD and PAH. VIP has been shown to exert immunomodulating and anti-inflammatory activities through VIP specific receptors [74]. VIP inhibits the release of mediators from pulmonary mast cells, interacts with T lymphocytes, prevents lung injury due to xanthine oxidase and may act as a free radical scavenger [75-78]. VIP also inhibits the production of IL-6, IL-12, TNF alpha, and nitric oxide, and stimulates IL-10 production, and these effects are mostly mediated through the constitutively expressed VPAC1 receptor at the transcriptional level via modulation of NFκB and cAMP responsive element (CRE)-binding or ets-2 complexes [79]. Dunzendorfer *et al. *have suggested that VIP has an anti-inflammatory effect on eosinophils, reporting that VIP inhibited eosinophil migration and production of IL-16 *in vitro*, which subsequently inhibited chemotaxis of lymphocytes [80,81]. Delgado *et al. *also reported that VIP inhibited LPS-induced inflammatory pathways in monocytes and macrophages via cAMP-dependent or independent mechanisms [55]. In addition, it has been suggested that VIP functions as an important T helper-differentiating factor that promotes Th2-like and inhibits Th1-like immune response via several mechanisms, including preferential survival of Th2 effectors and generation of memory Th2 cells [82]. In vitro studies show that VIP treatment leads to the induction of IL-4 and IL-5 in macrophages, and leads to the inhibition of IFN-gamma and IL-2 in antigen-primed CD4 T cells [83]. Mice lacking VPAC2 showed increased Th1-type responses which were characterized by an enhanced delayed type hypersensitivity and a diminished immediate-type hypersensitivity [58] In contrast, T cell over-expression of VPAC2 led to a deviation from the normal CD4 T cell cytokine expression profile toward a Th2-like profile with elevated blood IgE and IgG1 levels and increased eosinophil numbers. These transgenic mice also showed increased cutaneous allergic reactions, and a decreased delayed-type hypersensitivity [58]. Future study should further examine the immune-regulatory role of VIP using animal models with T cell-related diseases such as allergic asthma.

## 8. Therapeutic potential of VIP in PAH

The main pathological features of PAH in the pulmonary vasculature are perivascular inflammation, thrombosis, abnormal growth of vascular smooth muscle cells and extracellular matrix accumulation, leading to remodeling of the pulmonary vessel wall, obstruct pulmonary blood flow and ultimately cause right heart failure. Current treatment of PAH, which includes the use of prostacyclins, endothelin receptor antagonists, and phosphodiesterase type 5 inhibitors, either alone or in combination, have only limited efficacy in the improvement of clinical symptoms, hemodynamics, and long-term survival [12-14]. VIP has a large spectrum of biological functions including potent dilatory actions in pulmonary blood vessels and airway smooth muscles, potent anti-inflammatory actions, inhibition of vascular smooth muscle cell proliferation, enhancing would healing, regulation of cell growth and survival, and modulation of airway secretions. Therefore, using VIP-based drugs to target the various components of this multifactorial disease could be a novel therapeutic approach for the treatment of PAH.

In monocrotaline-induced pulmonary hypertension in rabbits, VIP dose-dependently decreased pulmonary artery pressure and pulmonary vascular resistance [83]. Application of VIP to patients with primary pulmonary hypertension results in substantial improvement of hemodynamic and prognostic parameters of the disease without side effects [36]. It decreased the mean pulmonary artery pressure in these patients, increased cardiac output, and mixed- venous oxygen saturation [36]. Said indicated that VIP gene is a key modulator of pulmonary vascular remodeling and inflammation [84]. Mice lacking VIP gene developed moderately severe PAH, with right ventricular hypertrophy, and thickened pulmonary artery, as well as perivascular inflammatory cell infiltrates in the lung [85]. Treatment of the mice with VIP attenuated both the vascular remodeling and right ventricular remodeling [85]. Right heart failure is a hallmark of severe PAH, and ultimately leading to death. In animals and in humans, infusion of VIP increases the epicardial coronary artery cross-sectional area by 27%, decreases coronary vascular resistance by 46%, and increases coronary artery blood flow by 200% [20,86]. Application of VIP to patients also increases the left ventricular fraction shortening by 38% and significantly increases left ventricular contractility [86,87]. Therefore, addition to its actions on decreasing pulmonary artery pressure, VIP also protects the heart.

## 9. Therapeutic potential of VIP in COPD/asthma

Chronic inflammatory airway diseases such as COPD and bronchial asthma continue to be an important cause of morbidity, mortality, and health-care cost worldwide. The key clinical features of asthma are airflow obstruction and airway hyperresponsiveness that caused by airway inflammation [16]. Many of the inflammatory events in asthma are thought to be mediated by Th2 cells. It also involves mast cells, eosinophils, neutrophils and mesenchymal cells such as epithelial cells, fibroblasts, smooth muscle cells and endothelial cells. The inflammatory mediators, including cytokines, chemokines, adhesion molecules, proteinases and growth factors released by these cells participant in this process at various stages and interact to maintain and amplify the inflammatory response [11]. Two categories of drugs are currently used in asthma therapies: bronchodilators and anti-inflammatory drugs. Despite the availability of these medications, the asthma epidemic continues to increase. The key clinical feature of COPD is airflow limitation results from airway constriction and irreversible reduction in the caliber of the small airways of the lung. Cigarette smoking is an important risk factor of COPD. The airflow limitation or obstruction that happens in COPD is caused by a mixture of small airway disease, parenchymal destruction (emphysema) and in many cases, increased airway responsiveness (asthma) [15]. Studies have shown that there is a large overlap of up to 30% between people who have a clinical diagnosis of COPD and asthma [88]. There is also a high incidence of mild to moderate PAH prevalence, reaching to 50% in advanced chronic obstructive COPD [89]. As Said suggested that PAH/asthma/COPD share important pathological features, including inflammation, smooth muscle contraction and remodeling [90]. Inflammation has long been acknowledged as a key feature of the asthma and COPD [11,15,16,88,89]. Perivascular inflammation has also been increasingly recognized as a significant component of clinical and experimental PAH phenotypes [91]. In these diseases there is increased resistance in, and narrowing of, airways and pulmonary arteries, respectively, due to airway and pulmonary vasoconstriction, smooth muscle constriction, and thickening of the walls caused by smooth muscle and other cell proliferation known as remodeling [90]. Muscularisation and remodeling of smaller pulmonary arteries are essential pathological lesions in PAH [92]. Airway remodeling caused by airway inflammation includes an increase in airway wall thickness, fibrosis, smooth muscle mass and vascularity, as well as abnormalities in extracellular matrix composition [89,93]. These shared pathological features suggest possible common underlying mechanism among PAH/asthma/COPD.

Mice with targeted deletion of VIP gene, simultaneously express airway hyperresponsiveness with airway inflammation, together with PAH, pulmonary vascular remodeling and perivascular inflammation. Treatment of the mice with VIP reversed both sets of phenotypic changes, confirming that they result from the absence of the VIP gene [10,84]. Recently, attention has been drawn to the therapeutic potential of VIP for the clinical treatment of COPD/asthma on the basis that VIP acts as a neurotransmitter, the dominant mechanism of human airway and vascular relaxation, and its anti-inflammatory properties. Neutrophil accumulation in the airway is a characteristic feature of COPD and asthma. VIP and its analogues have been shown to inhibit antigen- or cytokine-induced neutrophil recruitment in the airway *in vivo *[94]. VIP has also been shown to attenuate the cigarette smoke extract-induced apoptotic death of rat alveolar L2 cells, and protect against human bronchial epithelial cell damage, enhance airway wound healing [95,96]. Recent studies show that inhalable powder formulation of VIP derivative, IK312532 attenuates airway inflammation in ovalbumin challenge-induced asthma/COPD -like rats and in cigarette smoke-exposed rats [9,97,98].

## 10. VIP for clinical use

The key to the therapeutic use of VIP in human disease is in its delivery. Firstly, VIP is degraded quickly by enzymes, catalytic antibodies, and spontaneous hydrolysis in biological fluids. Secondly, systemic administration of VIP has been shown to cause cardiovascular side effects [59]. To overcome the limited clinical effectiveness of native VIP, VIP incorporated into phospholipids has been used successfully in animal models of pulmonary hypertension [99]. Furthermore, several peptidase-resistant VIP-analogues have been developed [100]. VIP analogue, Ro 25-1553 causes a concentration-dependent relaxation of airway and pulmonary artery preparations, with an EC50 of approximately 10 nM and a maximal relaxation of 70%-75% of the induced tone [101]. In patients with asthma, inhalation of a selective VPAC2 receptor agonist Ro 25-1553 causes a bronchodilatory effect. The corresponding maximum bronchodilatory effect during 24 hours was similar for Ro 25-1553 and the reference bronchodilator formoterol (beta-2 adrenoceptor agonist). However, the bronchodilatory effect of Ro 25-1553 was attenuated 5 hours after inhalation whereas formoterol still had a bronchodilatory effect 12 hours after inhalation [102]. Therefore, the development of effective drug delivery systems for VIP-based respiratory therapy remains a significant challenge. It is possible to envisage that development of controlled-release biodegradable VIP-based drug system, particularly with airway delivery capability would have very significant therapeutic benefits in the treatment of cardiopulmonary diseases, including PAH, COPD and asthma.

## 11. Conclusion

This article describes the physiological significance of VIP and its therapeutic potential for the treatment of cardiopulmonary diseases, including PAH, asthma, and COPD. VIP exerts a variety of actions, including potent dilatory actions in pulmonary blood vessels and airway smooth muscles, potent anti-inflammatory and anti-proliferative actions, regulation of cell growth and survival, and modulation of airway secretions. PAH, asthma and COPD share key mechanisms of pathogenesis, including inflammation, smooth muscle contraction and remodeling. No other existing or potential drug provides the combined potential advantages of lowering pulmonary arterial pressure, reducing bronchoconstriction, improving blood circulation to the heart and lung, reducing inflammation of the heart and lung tissues, and enhancing wound healing of bronchial epithelial cells. Therefore, development of drug delivery system for VIP-based respiratory therapy may be a promising strategy for the treatment of PAH, asthma and COPD.

## List of abbreviations

VIP: vasoactive intestinal peptide; VIP-IR: VIP-immunoreactivity; PAH: pulmonary arterial hypertension; COPD: chronic obstructive pulmonary disease; PACAP: pituitary adenylate cyclase activating peptide; VPAC1: VIP/PACAP receptor type1; VPAC2: VIP/PACAP receptor type 2; NOS: nitric oxide synthase; CRE: cAMP responsive element.

## Competing interests

The authors declare that they have no competing interests.

## Authors' contributions

All authors participated in drafting the manuscript. All authors read and approved the manuscript.
